# Gold-Conjugated Nanobodies for Targeted Imaging Using High-Resolution Secondary Ion Mass Spectrometry

**DOI:** 10.3390/nano11071797

**Published:** 2021-07-10

**Authors:** Paola Agüi-Gonzalez, Tal M. Dankovich, Silvio O. Rizzoli, Nhu T. N. Phan

**Affiliations:** 1 Center for Biostructural Imaging of Neurodegeneration, University Medical Center Göttingen, von-Siebold-Straße 3a, 37075 Göttingen, Germany; paola.aguigonzalez@med.uni-goettingen.de (P.A.-G.); srizzol@gwdg.de (S.O.R.); 2 Department of Neuro- and Sensory Physiology, University Medical Center Göttingen, Humboldtallee 23, 37073 Göttingen, Germany; tal.dankovich@med.uni-goettingen.de; 3 Department of Chemistry and Molecular Biology, University of Gothenburg, Kemivägen 10, 41296 Gothenburg, Sweden

**Keywords:** nanobodies, gold nanoparticles, target imaging, NanoSIMS, SIMS

## Abstract

Nanoscale imaging with the ability to identify cellular organelles and protein complexes has been a highly challenging subject in the secondary ion mass spectrometry (SIMS) of biological samples. This is because only a few isotopic tags can be used successfully to target specific proteins or organelles. To address this, we generated gold nanoprobes, in which gold nanoparticles are conjugated to nanobodies. The nanoprobes were well suited for specific molecular imaging using NanoSIMS at subcellular resolution. They were demonstrated to be highly selective to different proteins of interest and sufficiently sensitive for SIMS detection. The nanoprobes offer the possibility of correlating the investigation of cellular isotopic turnover to the positions of specific proteins and organelles, thereby enabling an understanding of functional and structural relations that are currently obscure.

## 1. Introduction

Nanoscale secondary ion mass spectrometry (NanoSIMS), an imaging technique characterized by high spatial resolution and high sensitivity, has become a valuable analytical tool for molecular imaging in biological research [1]. The technique enables the analysis of multiple analytes in a single measurement at subcellular resolution, providing a better understanding of a complex interaction of cellular molecules and structures. Across the broad range of achievable lateral resolutions with SIMS instruments, NanoSIMS particularly allows the detection of elemental and small ion species at a spatial resolution down to 50 nm. However, due to the trade-offs with sensitivity for the detection of analytes in biological samples, a lateral resolution of ~100 nm is common for most experiments [2]. The lateral resolution achieved with NanoSIMS is relatively comparable to super-resolution light microscopy and electron microscopy techniques. However, in general, SIMS exhibits a drawback: it is difficult to identify cellular structures and localize specific proteins, which are necessary for an insight into the relationship between molecular organization and function at a subcellular level. In SIMS, the spatial resolution depends both on the type of the primary ion beam and the abundance of the analytes. NanoSIMS is certainly capable of subcellular imaging. In ToF-SIMS, with a bismuth liquid metal ion gun, a spatial resolution down to 500 nm could be achieved. With a C_60_^+^ gun, a spatial resolution of approximately 1 µm could be routinely achieved. With a gas cluster ion beam (GCIB) such as (Ar)_n_^+^ GCIB, (H_2_O)_n_^+^ GCIB, the obtained lateral resolution could be several µm. The size of normal cells is usually at least 10 µm, which means that ToF-SIMS is suitable for subcellular imaging. Examples of subcellular imaging with ToF-SIMS can be found in the representative literature [3,4,5,6,7].

A common approach to complement the information of morphology or protein localization is to correlate SIMS with other imaging techniques such as electron microscopy, fluorescence microscopy, or matrix-assisted laser desorption ionization (MALDI) [8,9,10]. Correlative imaging offers the means to connect different properties, structures, and functions of the cells. However, it is highly challenging due to the compatibility requirements of the instrumentation and sample preparation. An alternative approach is to employ probes to label specific proteins, which are then detectable by SIMS. This is a more straightforward solution, alleviating the complicated nature of the correlation method. Target molecular imaging with NanoSIMS and SIMS has been a long-researched topic in the field. Up to now, labeling probes for specific proteins have been available to a very limited extent due to their highly challenging development process. A suitable probe must satisfy several requirements. It should contain elements that are easily ionized and rarely present in biological specimens, and it should selectively bind to the target molecules with sufficient density to be detectable in SIMS. A few labeling probes were recently developed and successfully applied to image different proteins in cells at nanoscale resolution—for example, dual probes containing isotopic element boron, or fluorine, and a fluorophore for SIMS and fluorescence microscopy imaging [11,12,13]. Such small-sized probes are beneficial to SIMS imaging regarding the imaging resolution, labeling precision, and labeling density. However, they often exhibit difficulty detecting low-abundance molecules due to the high background signal level, which comes from the embedding resin and the substrate materials commonly used for NanoSIMS and SIMS experiments, particularly silicon wafers.

Labeling probes containing metal coupled to antibodies that can be detected by NanoSIMS and EM have been employed for imaging biological samples [14,15,16]. For example, colloidal gold-coupled antibodies were used to image actin and synaptophysin in mammalian cells [17]. In this case, the tissue was fixed, embedded in LR White plastic resin, and cut into sections that were subsequently immunostained with a colloidal gold-coupled antibody. This post-embedding staining approach often encounters an issue wherein many epitopes in the sample are not revealed due to the limited ability of the antibody to penetrate into the resin-embedded sections. In addition, the chemical fixation and tissue preparation before the embedding are often not optimal for antigen preservation, which results in low detection of the signals of the molecules of interest. To increase the epitope accessibility in the embedded samples, optimizations for the conditions of resin polymerization or a modification of the resin-embedded samples, such as resin etching with Na-ethanolate and antigen retrieval by sodium dodecyl sulfate at 0.5%, were reported [18,19].

Lanthanides were coupled to antibodies for detecting multiple proteins in human breast cancer tissues using NanoSIMS [20]. Nevertheless, the large size of the antibodies could be a limiting factor for the spatial resolution of NanoSIMS imaging due to the possible clustering of the antibodies. The clustering also restricts the accessibility of the probes to all the epitopes, which could hinder the detectability of the molecules [21]. Lanthanides and gold have been commonly used for imaging in biological and medical studies due to their biocompatibility [22,23,24]. In particular, gold nanomaterials have been extensively used in mass spectrometry imaging because of their well-established synthetic procedures, which enable fully customized gold nanomaterials [22]. For SIMS imaging, the secondary ion yield heavily depends on the ionization energy for positive ions and the electron affinity for negative ions. Due to their low ionization energy [25], lanthanides are easily ionized and thus are more suitable for detection in positive ion mode [20]. On the other hand, gold is one of the six elements with the highest electron affinity (only after chlorine, fluorine, bromine, iodine, and astatine) [26]; therefore it is well suited for detection in negative ion mode.

In this study, we tested nanoprobes consisting of gold nanoparticles (Au NPs) conjugated to specific nanobodies to reveal specific proteins using NanoSIMS imaging. The nanobodies are very small in size (~3 nm in length) and thus are more compatible with NanoSIMS imaging at nanoscale resolution. In addition, the use of nanobodies helps to reduce the risk of clustering and epitope inaccessibility possibly caused by the antibodies [21,27,28]. Two types of nanoprobes were used. The first one consisted of a nanobody that can bind directly and specifically to endogenous proteins such as the vesicular glutamate transporter 1 (Au anti-vGlut1 nanobody). The second type of Au nanoprobe makes use of a nanobody that can bind specifically to mouse immunoglobulins (Au anti-mouse secondary nanobody). We employed these nanoprobes to label different proteins in primary cultures of rat hippocampal neurons to demonstrate their potential for targeted bio-imaging with NanoSIMS.

## 2. Materials and Methods

### 2.1. Coupling Procedure for Au NPs–Anti-Mouse Secondary Nanobody

The reactive anti-mouse nanobody bearing 1 ectopic cysteine (N1202; NanoTag Biotechnologies) was used for the coupling reaction with 3 nm mono-maleimide Au NPs (C11-3 MMAl-DRY-2.5; Nanopartz Inc., Loveland, CO, USA). The coupling was performed similarly as previously described [11,12]. In brief, ~30 nmol of the nanobody containing C-terminal extra cysteine was reduced using 10 mM Tris(2-carboxyethyl)phosphine hydrochloride (TCEP) in PBS at pH~7 for 1 h on ice. Excess of TCEP was then removed using a NAP5 column (GE Healthcare) equilibrated with ice-cold and degassed PBS at pH~7. The reduced nanobody was mixed immediately with ~50 nmol of thiol reactive Au NPs. The reaction was left for 2 h on ice. Coupled nanobodies were purified using an Äkta HPLC system and a size-exclusion column (Superdex 75 increase, 10/300 GL). A chromatograph of the purified Au–anti-mouse nanobody conjugation is shown in Figure S1.

The anti-vGlut1 nanobody (N1602, NanoTag Biotechnologies, Göttingen, Germany), conjugated to mono-maleimide 1.4 nm colloidal gold from Nanoprobes, Inc. (Yaphank, NY, USA), was purchased as a custom product from NanoTag Biotechnologies GmbH (Göttingen, Germany).

### 2.2. Cell Culture and Preparation

Hippocampal neurons isolated from rat brains (P0) were seeded on silicon wafers and cultured in N2 medium at 37 °C in a humid atmosphere with CO_2_ 5% for two weeks. To prepare for experiments, neuronal cells were fixed with paraformaldehyde (PFA) 4% in PBS for 30 min at RT, quenched with glycine 100 mM in PBS, permeabilized, and blocked with a permeabilizing/blocking solution containing BSA 2.5% and Triton X-100 0.1% in PBS for 1 h at RT.

#### 2.2.1. Direct Immunostaining

After being fixed, quenched, blocked, and permeabilized, the cells on the silicon wafers were incubated with the Au anti-vGlut1 nanobody in the permeabilizing/blocking solution at RT for 1 h, followed by washing with BSA 2.5% in PBS for 3 × 5 min. The labeled cells were then washed sequentially with PBS, high-salt PBS (NaCl 362 mM in PBS, pH 7.4), PBS, and MQ water, followed by air-drying before NanoSIMS imaging.

#### 2.2.2. Indirect Immunostaining

After being fixed, quenched, blocked, and permeabilized, the cells on the silicon wafers were incubated with the mouse antibody of the protein of interest (POI), specifically a synaptic protein Synaptotagmin 1 (SySy, 105 311) or a mitochondrial marker TOM20 (Merck KGaA, Darmstadt, Germany, WH0009804M1), in the permeabilizing/blocking solution at RT for 1 h, followed by washing with BSA 2.5% in PBS for 3 × 5 min. Afterward, cells were incubated with the Au anti-mouse secondary nanobody in the permeabilizing/blocking solution at RT for 1 h, followed by washing with BSA 2.5% in PBS for 3 × 5 min. The labeled cells were then washed sequentially with PBS, high-salt PBS (NaCl 362 mM in PBS, pH 7.4), PBS, and MQ water followed by air-drying before NanoSIMS imaging.

### 2.3. Confocal Microscopic Imaging

For the confocal imaging experiments, hippocampal neurons were plated on glass coverslips. Following the same protocol as for the samples imaged with NanoSIMS, the neurons were first fixed and then immunostained. For comparison with the NanoSIMS results observed from the anti-Vglut1 gold-conjugated nanobody, we incubated the neurons with a FluoTag-X2 anti-vGlut1 nanobody directly conjugated to STAR580 (NanoTag, N1602), for 1 h at RT, in blocking solution. Likewise, to validate the results obtained with SIMS using the anti-mouse gold-conjugated secondary nanobody, we incubated the hippocampal neurons with the same mouse anti-TOM20 primary antibody, and with FluoTag-X2 anti-mouse secondary nanobodies conjugated to STAR635P (NanoTag, N2002), for 1 h at RT, in blocking solution. Following the incubations, the neurons were washed with PBS and then embedded in Mowiol. Confocal imaging was performed on an Abberior QUAD scan STED/confocal microscope (Abberior GmbH, Göttingen, Germany) equipped with a UPlanSApo 100 × 1.4 NA objective (Olympus Corporation, Shinjuku, Tokyo, Japan) and an EMCCD iXon Ultra camera (Andor, Belfast, Northern Ireland, UK). The samples were excited using pulsed 485 nm, 580 nm, and 640 nm lasers for imaging the autofluorescence background signal and the proteins of interest, vGlut1 and TOM20, respectively. The pinhole size was set to 1 airy unit.

### 2.4. NanoSIMS Imaging and Data Analysis

The NanoSIMS imaging was carried out by a NanoSIMS 50 L (Cameca, Grennevilliers, France) with an 8 kV cesium primary ion source in negative ion mode. A primary ion current of 0.5–1 pA (D1-3 or D1-4) was used to sputter sample areas of 25–30 µm to obtain images of 256 × 256 pixels or 512 × 512 pixels for imaging vGlut and TOM20. For Synaptotagmin, the primary ion current of ~15 pA (D1-1) was used. The sample was first implanted at the primary ion current ~110 pA (D1-0) with the primary ion dose of ~1.1 × 10^16^ ion/cm^2^ to obtain a steady state of the sputter rate and ionization before imaging. To ensure a sufficient mass resolution to separate isobaric mass peaks, an entrance slit of 20 × 140 µm (ES:3) and an aperture slit of 300 × 300 µm (AS:1) were used. All the NanoSIMS images were obtained from five consecutive layers, each with a dwell time of 5000 µs/pixel.

The analyzed ions were ^12^C^14^N^−^, ^197^Au^−^, and ^28^Si_2_^−^, which are expressed as ^12^C^14^N, ^197^Au, and ^28^Si_2_ in the paper. ^12^C^14^N is a common fragmented ion from biomolecules such as proteins, DNA, RNA, etc.; therefore, it is often used to locate the cell areas and to provide an overview of the sample topography. Likewise, ^197^Au is detected to locate the gold nanoprobes. ^28^Si_2_ is from the Si substrate on which the cells are placed.

NanoSIMS image exportation, drift correction if necessary, stacking from individual image layers, line profile measurement, and image ratio measurement (^197^Au/^12^C^14^N) were carried out using the NanoSIMS analysis software (version 4.5, Cameca, Grennevilliers, France) and the OpenMIMS plugin, ImageJ (NRIMS, Cambridge, MA, USA) [29]. To compare the ^197^Au/^12^C^14^N ratio signal between samples labeled with Au nanoprobes and negative control samples (labeled with Au nanoprobe in the absence of primary antibody, and labeled with neither primary antibody nor Au nanoprobe), self-written MATLAB (R2016b, the MathWorks Inc, Natick, Massachusetts, MA, USA) scripts were used. To compare the ^197^Au/^12^C^14^N ratio between regions of interest (ROI) of different samples, circular ROIs with a diameter of 5 pixels were manually selected, as shown in Figure 2 and Figure S2A. In total, 35 ROIs were selected from the cells labeled with Au anti-vGlut1 nanobody, and 10 ROIs were selected from non-labeled cells. For the comparison of the Au anti-mouse secondary nanobody and the respective controls, 15 ROIs were selected on the cells labeled with anti-TOM20 primary mouse antibody and Au anti-mouse secondary nanobody, 10 ROIs were selected from the cells labeled with Au anti-mouse secondary nanobody in the absence of the anti-TOM20 primary antibody, and 10 ROIs were selected from the cells that were labeled neither with the anti-TOM20 primary antibodies nor gold secondary nanobodies. From each pixel of the ROI, the ^197^Au/^12^C^14^N ratio was extracted and then was averaged across all pixels of the ROI. We then applied the Kolmogorov–Smirnov test to statistically compare the gold signal between the labeled and negative control samples.

## 3. Results

**Figure 1:**
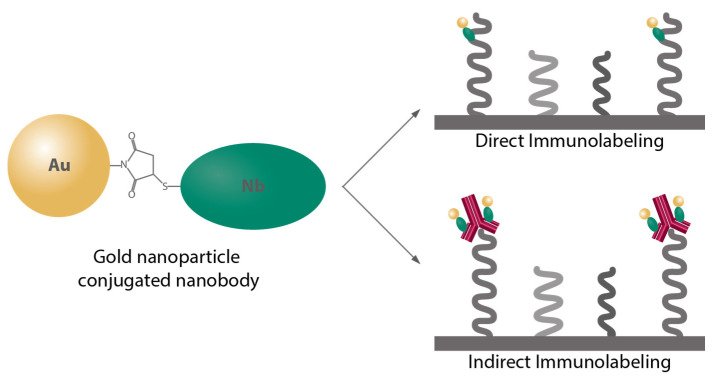
Au nanoprobes for revealing proteins of interest (POIs) via either direct or indirect immunolabeling strategies. Direct immunolabeling is obtained by using an Au nanoprobe detecting endogenous POIs. For indirect immunolabeling, the POIs are first recognized by a specific primary mouse antibody, which is then revealed by an Au anti-mouse secondary nanobody.

Au NPs with a functional group maleimide were conjugated to the nanobodies having the cysteine residues via the cysteine maleimide reaction. For the direct immunostaining approach, a nanobody against the endogenous protein in the cells was used, as demonstrated here, the Au NP-coupled anti-vGlut1 nanobody (Au anti-vGlut1 nanobody) for labeling the vesicular glutamate transporter 1. The indirect immunostaining was facilitated by the Au NPs coupled to the nanobody against a light chain of the mouse antibody (Au anti-mouse secondary nanobody), which allowed us to label any proteins of interest recognized beforehand with a mouse antibody. The labeling strategies using these Au nanoprobes are illustrated in Figure 1.

The Au nanoprobes were tested on different cellular proteins using NanoSIMS imaging. First, the Au anti-vGlut1 nanobody was used to immunostain the vGlut1 in fixed hippocampal neurons from rats. The cells were then embedded in LR White resin, cut into thin sections (200 nm), placed onto silicon wafers, and subsequently measured with NanoSIMS.

**Figure 2:**
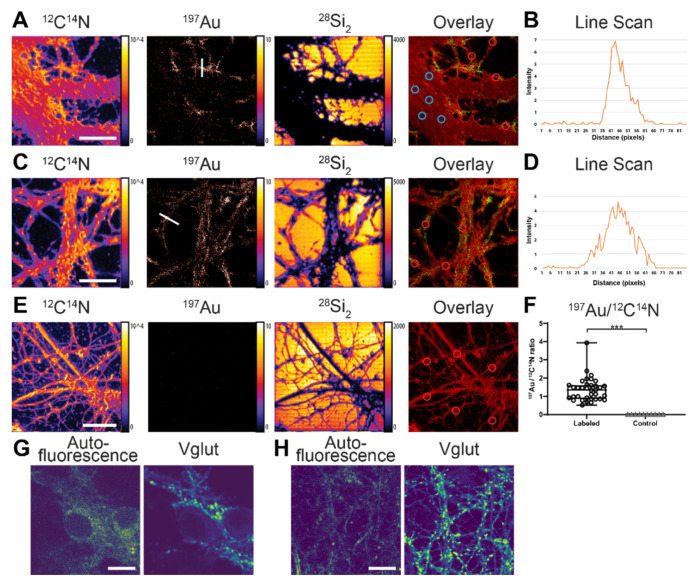
NanoSIMS imaging of vGlut protein in rat hippocampal neurons via direct immunolabeling with the Au anti-vGlut1 nanobody. (**A**) Cell body and neurites, 512 × 512 pixels. From left to right: ion images of ^12^C^14^N, ^197^Au, ^28^Si_2_, and overlay of ^197^Au (green) and ^12^C^14^N (red). The blue circles in the overlaid image indicate the cell body while the red circles indicate the neurite areas. (**B**) A line-scan profile of ^197^Au signal across a neurite in ^197^Au image (white line). (**C**) Neurites, 512 × 512 pixels. From left to right: ion images of ^12^C^14^N, ^197^Au, ^28^Si_2_, and overlay of ^197^Au (green) and ^12^C^14^N (red). The red circles in the overlaid image indicate the neurite areas. (**D**) A line-scan profile of ^197^Au signal across a neurite in ^197^Au image (white line). (**E**) Negative control cell without Au anti-vGlut1 nanobody. From left to right: ion images of ^12^C^14^N, ^197^Au, ^28^Si_2_, and overlay of ^197^Au (green) and ^12^C^14^N (red). (**F**) Comparison of signal intensity of ^197^Au between the cells labeled with the Au anti-vGlut1 nanobody and the negative control cells. Significantly higher signal in the labeled cells compared to the control cells verified by the Kolmogorov–Smirnov test (*** *p* < 0.0001). Each data point represents the average value of ^197^Au/^12^C^14^N ratio from all the pixels of individual ROIs (*n* = 35 for labeled cells, *n* = 10 for control cells). Error bars show the SEM. Color scale of ^197^Au image is scaled to the maximum intensities of respective ^12^C^14^N image. (**G**,**H**) Exemplary confocal fluorescence images of vGlut1 in rat hippocampal neurons revealed by STAR580 conjugated anti-vGlut1 nanobodies (*n* = 10 images). (**G**) vGlut1 localizes predominantly to the neurites, as compared with the cell body. (**H**) vGlut1 localizes to “hot spots” along the neurites. Scale bars are 10 µm.

NanoSIMS images were recorded simultaneously for several ions, particularly ^12^C^14^N, ^28^Si_2_, and ^197^Au. The ion image of ^197^Au showed that the ^197^Au signal was considerably higher in the neurite area compared to the cell body (Figure 2A). The ^12^C^14^N image showed that the ^12^C^14^N signal was not very homogenous due to some topographical differences across the cell area (although they were not too severe). There was some signal enhancement at the edges due to this issue. Furthermore, the ^12^C^14^N ion images showed the opposite distribution to the ^28^Si_2_ signal, which was from the substrate. This means that the ^197^Au signal was obtained within the cell area where the ^28^Si_2_ did not cover.

The overlayed image of ^197^Au and ^12^C^14^N showed a distinct localization of the nanobody in the neurite area. In particular, the ^197^Au signal was localized as “hot spots” along the neurites, as expected for the distribution of vGlut in neurons [30,31] (Figure 2C). The line scan profiles across the “hot spot” in the neurites showed significant signal-to-noise of the ^197^Au signal for the labeled structure (Figure 2B,D). In addition, the ^197^Au signal was significantly higher in the cells labeled with the Au anti-vGlut1 nanobody compared to the non-labeled cells according to the Kolmogorov–Smirnov test at *p* < 0.0001 (Figure 2F). To confirm the localization of the vGlut via the ^197^Au signal in NanoSIMS, we performed confocal fluorescence microscopic imaging of vGlut in similar neuronal cells. In this case, the vGlut was labeled with an anti-vGlut1 primary nanobody directly conjugated to the STAR580 fluorophore. An autofluorescence image of the entire cells was also included for comparing the general shape of the cells with the ^12^C^14^N image in NanoSIMS. A comparison between the NanoSIMS and confocal images showed a similar distribution of vGlut in the neurites of the cells (Figure 2G,H). The results showed that Au anti-vGlut1 nanobody was specific for the POI, selective, and sufficiently sensitive for detection by NanoSIMS. The imaging resolution of the vGlut1 structure calculated from a line-scan analysis was ~91 nm (Figure S3). This resolution of the NanoSIMS for vGlut is superior to that of the fluorescence confocal imaging (around 200–250 nm). However, fluorescence confocal microscopy has relatively high sensitivity. In principle, it can detect single fluorophores, although it is unlikely to achieve this in conventional samples without substantial optimization. Therefore, confocal images are often brighter and have a higher signal-to-noise ratio as compared to NanoSIMS images.

We then tested the second type of nanoprobe, Au anti-mouse secondary nanobody, for different proteins, including the synaptic protein Synaptotagmin 1 and the mitochondrial marker TOM20. The fixed neurons were first immunoassayed with a mouse primary antibody that recognized each POI, followed by the Au anti-mouse secondary nanobody. The labeled cells were then allowed to air dry before the NanoSIMS measurement.

**Figure 3:**
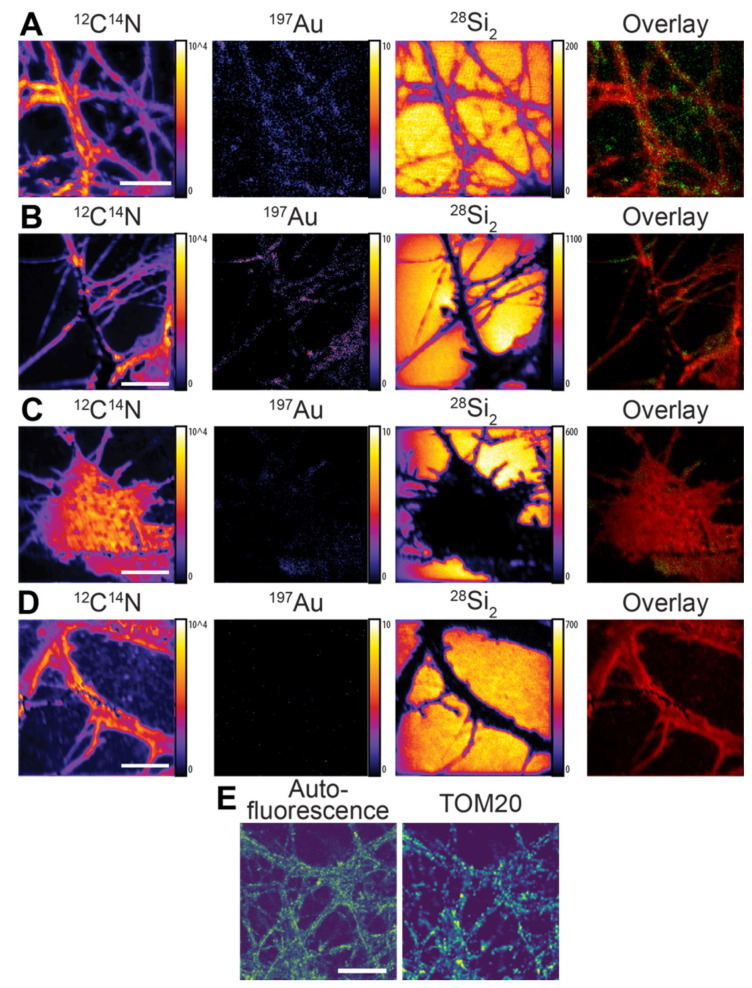
NanoSIMS imaging of specific proteins in hippocampal neurons via indirect immunolabeling with the Au anti-mouse secondary nanobody. (**A**) Synaptic protein Synaptotagmin 1. 128 × 128 pixels. From left to right: ion images of ^12^C^14^N, ^197^Au, ^28^Si_2_, and overlay of ^197^Au (green) and ^12^C^14^N (red). (**B**) Mitochondrial marker TOM20. 256 × 256 pixels. From left to right: ion images of ^12^C^14^N, ^197^Au, ^28^Si_2_, and overlay of ^197^Au (green) and ^12^C^14^N (red). (**C**) Negative control cell with Au anti-mouse secondary nanobody in the absence of primary antibody. 256 × 256 pixels. From left to right: ion images of ^12^C^14^N, ^197^Au, ^28^Si_2_, and overlay of ^197^Au (green) and ^12^C^14^N (red) (**D**) Negative control cell in the absence of both primary antibody and Au anti-mouse secondary nanobody. From left to right: ion images of ^12^C^14^N, ^197^Au, ^28^Si_2_, and overlay of ^197^Au (green) and ^12^C^14^N (red). Color scale of ^197^Au image is scaled to the maximum intensities of respective ^12^C^14^N image. (**E**) Exemplary confocal images of mitochondrial marker TOM20 in rat hippocampal neurons tagged with the anti-TOM20 primary mouse antibody. The primary antibody was revealed by STAR635P-conjugated anti-mouse secondary nanobodies (*n* = 10 images). Scale bars are 10 µm.

The ^12^C^14^N signal was not evenly distributed across the NanoSIMS images, which could account for the low degree of sample topography (Figure 3) due to the use of the whole dried cells for imaging. For all the samples, the distribution of ^12^C^14^N was opposite to that of the ^28^Si_2_, meaning that the gold signal was obtained within the cell area that the ^28^Si_2_ did not cover. For the Synaptotagmin 1 labeling, the ^197^Au signal localized as small speckles along the neurites, which is in good agreement with other studies [32,33] (Figure 3A). The labeling of mitochondrial marker TOM20 showed a distribution in the cytoplasm across the cells [34] (Figure 3B). In the negative control cells labeled with the Au anti-mouse secondary nanobody in the absence of the primary antibody, there was a low level of ^197^Au signal, which was possibly caused by the small extent of non-specific binding of the nanobody (Figure 3C). However, the ^197^Au signal in the control samples was significantly lower compared to that in the labeled cells. Furthermore, a negligible signal of ^197^Au was detected in the negative control cells labeled with neither the primary antibody nor the Au anti-mouse secondary nanobody (Figure 3D). The labeled cells showed a statistically higher ^197^Au signal compared to those of the negative control cells based on the Kolmogorov–Smirnov test (*p* < 0.0001) (Figure S2B). The distribution of TOM20 in the NanoSIMS images showed a similar pattern to that observed in the confocal fluorescence images of TOM20, where the antibody location was revealed by an anti-mouse secondary nanobody conjugated to the STAR635P fluorophore (Figure 3E). The Au anti-mouse secondary nanobody was shown to have sufficient selectivity to the corresponding primary antibody, high flexibility for the two examined target proteins, and sufficient sensitivity for the NanoSIMS measurement. The imaging resolution of the structures of TOM20 and Synaptotagmin 1 calculated from a line-scan analysis was approximately 116 nm and 981 nm, respectively (Figure S3). Synaptotagmin 1 had a poorer resolution compared to TOM20 because a larger diaphragm D1-1 was used to increase the detected signal for Synaptotagmin 1.

## 4. Discussion

We have previously developed several dual-labeling probes for the detection of specific cellular proteins. Each probe had a fluorophore and an isotopic element, such as boron [11] or fluorine [12], allowing imaging with both fluorescence microscopy and NanoSIMS on the same sample. These probes showed high performance in terms of imaging resolution, labeling precision, and labeling density. However, they could encounter problems associated with the high background signal from the embedding resin and the substrate materials. In this work, we generated a gold anti-mouse secondary nanobody and demonstrated its applications, in parallel with a custom-made product, the Au anti-vGlut1 nanobody, for immunolabeling various cellular proteins and imaging with NanoSIMS. The Au NPs provide a high signal-to-background in SIMS as they are rarely present in biological and embedding materials. Immuno-detection is a straightforward approach, allowing the nanoprobes to reveal the endogenous proteins without any genetic manipulation. There are advantages and disadvantages of using a conjugated nanobody or conjugated antibody. While the nanobody can penetrate epitopes better due to its small size compared to an antibody, such Au particles linked to the antibody [17] would result in a higher amount of Au atoms per epitope, and thus higher sensitivity for SIMS measurement, compared to the nanobody, since multiple particles are linked to each antibody. However, the conjugation of Au particles to the antibody is less reproducible than the procedure for the nanobody. The reason is that the nanobody has comparatively few amino acid side chains, so it can be linked to the gold particles at specific sites, thereby ensuring that every single batch of nanobody is conjugated in the same fashion [35]. On the other hand, an antibody is much larger and has more amino acid side chains, and its conjugation to various labels is difficult to standardize. While modern molecular biology approaches enable the cloning and manipulation of IgGs, and their site-specific labeling, the wide majority of academic and commercial laboratories still employ non-specific methods to link labels, as the Au particles to different side chains on each antibody, leading to large variability from batch to batch of the conjugation. 

In this study, nanobodies were conjugated to gold NPs using maleimide chemistry. The maleimide chemistry is expected to insignificantly affect the affinity of the nanobody, since the complementarity-determining regions (CDRs) of the nanobody are “far” from the C-terminus where the ectopic cysteine is used for conjugation with a maleimide. Regardless, the affinity is slightly modified; our data from NanoSIMS and confocal imaging clearly showed that the functional probe bound to its target with sufficient affinity. The maleimide conjugation on nanobodies was characterized in the work by Pleiner et al. [35].

The Au anti-vGlut1 nanobody allows direct binding of the POI vGlut, which offers high specificity, high labeling precision, and imaging at nanoscale resolution. The direct labeling nanoprobe, however, cannot be flexibly used for many proteins, but different proteins require different Au-coupled nanobodies to specifically recognize their epitopes. With the experiments using the Au anti-vGlut1 nanobody, we provided a proof of principle that Au-conjugated primary nanobodies exhibit specific, sufficient labeling for imaging a specific cellular protein, and, therefore, this approach is suitable for biological materials. Further work employing the Au-conjugated primary nanobodies on different POIs would be desirable. 

In addition, the palette of available nanobodies recognizing endogenous proteins is currently very limited. This limitation can be alleviated by the Au anti-mouse secondary nanobody, which enables a flexible application of the nanoprobe to any POI labeled with a primary mouse antibody. This more adaptable probe shows that NanoSIMS experiments can be performed using conventional antibodies, while still taking advantage of easy and efficient gold labeling with secondary nanobodies. From our results, the indirect labeling nanoprobe was shown to exhibit high selectivity and sensitivity for targeted molecular imaging with NanoSIMS. Moreover, this type of probe also allows the amplification in which multiple steps of labeling using different primary antibodies are performed in order to increase the detectability of low-abundance proteins on NanoSIMS. However, this could affect the labeling precision because the ^197^Au signal is placed far away from the epitopes. Further development of the nanoprobes is highly desirable to increase the sensitivity for SIMS detection by increasing the labeling sites of Au NPs on each nanobody alternatively to the multiple labeling approach. However, this needs a careful design to avoid the risk of precipitation caused by an increased amount of Au NPs per nanobody.

The gold nanoprobes are also applicable to other SIMS techniques such as ToF-SIMS, allowing the detection of various molecules, including metabolites, lipids, and small peptides, in relation to the localization of specific cellular structures labeled with the Au nanoprobes. As ToF-SIMS is well known as a label-free technique, labeling has not been commonly applied in this field. However, ToF-SIMS has a limited m/z range of detection in which large molecules such as proteins cannot be analyzed. This can be complemented by employing labeling probes for specific peptides and proteins [36,37,38]. For example, one application of antibody in ToF-SIMS imaging is the use of antibody-coupled liposomes for the simultaneous detection of amyloid-β, a main component of plaques in Alzheimer’s disease (AD), and lipids in the AD mouse brain [36]. Another example is multiplexed dynamic ToF-SIMS imaging for the characterization of different tumor microenvironments in tumors using 15 antibodies, each coupled with specific metal isotopes [38]. These studies demonstrate the value of implementing the information in ToF-SIMS to study a complex interaction of different molecules in diseases such as neurodegeneration and cancer. Nevertheless, these antibodies could only be used for labeling specific proteins (direct labeling approach). In our case, the Au anti-mouse secondary nanobody provides flexibility in labeling different proteins of interest. The monovalent nature and small size of the nanobody used for the nanoprobes eliminate the risk of clustering. In addition, the Au element can be detected with higher sensitivity by ToF-SIMS compared to other probes containing light elements such as boron or fluorine [11,12].

The gold nanoprobes would enable the correlation of NanoSIMS and EM imaging to obtain multidimensional information of the samples, which cannot be obtained by the individual techniques, particularly the turnover of the isotopic molecules and the localization of the protein architecture on NanoSIMS and its morphological property on EM.

## 5. Conclusions

We have successfully applied Au nanoprobes, which contain Au NPs conjugated to nanobodies, to image different target proteins in hippocampal neurons using NanoSIMS. The nanoprobes were shown to be well suited for SIMS imaging at subcellular resolution, with sufficient sensitivity and high specificity to the proteins of interest. The direct and indirect labeling of nanoprobes enables flexible immunostaining for a broad range of proteins to be imaged by NanoSIMS. The nanoprobes can also be used for other SIMS techniques, allowing the simultaneous imaging of specific cellular proteins and various analytes, including small molecules, metabolites, and lipids. Furthermore, the Au nanoprobes offer the possibility to correlate NanoSIMS and EM imaging to understand the functional and structural relationship at the subcellular level by combining the information of the cellular turnover, protein localization, and cell morphology.

## Data Availability

The data presented in this study are available on request to the corresponding author.

## Supplementary Materials

The following are available online at https://www.mdpi.com/article/10.3390/nano11071797/s1. Figure S1: Size-exclusion chromatograph of gold and anti-mouse nanobody conjugation. Figure S2: Representative region of interest (ROI) selection and a chart that compares ^197^Au signal in the neuronal cells labeled with TOM20 plus the Au anti-mouse secondary nanobody and in the negative control cells. Figure S3: Line-scans for determining the spatial resolution of NanoSIMS for vGlut1, TOM20, and Synaptotagmin 1.
