# Supplementary material for: Gold-Conjugated Nanobodies for Targeted Imaging Using High-Resolution Secondary Ion Mass Spectrometry
Source: Nanomaterials (Basel). 2021 Jul 10;11(7):1797. doi: 10.3390/nano11071797 (PMC8308316; doi:10.3390/nano11071797)
Supplement: Supplementary file 1 [file nanomaterials-11-01797-s001.zip › nanomaterials-1256183-supplementary.pdf]

Supplementary Materials

Gold-Conjugated Nanobodies for Targeted Imaging Using High-Resolution Secondary Ion Mass Spectrometry

Paola Agüi-Gonzalez [1,2], Tal M. Dankovich [2], Silvio O. Rizzoli [1,2] and Nhu T. N. Phan [1,2,3,*]

[1] Center for Biostructural Imaging of Neurodegeneration, University Medical Center Göttingen, von-Siebold-Straße 3a, 37075 Göttingen, Germany; paola.aguigonzalez@med.uni-goettingen.de (P.A.-G); srizzol@gwdg.de (S.O.R.)
[2] Department of Neuro- and Sensory Physiology, University Medical Center Göttingen, Humboldtallee 23, 37073 Göttingen, Germany; tal.dankovich@med.uni-goettingen.de
[3] Department of Chemistry and Molecular Biology, University of Gothenburg, Kemivägen 10, 41296 Gothenburg, Sweden
* Correspondence: nhu.phan@chem.gu.se

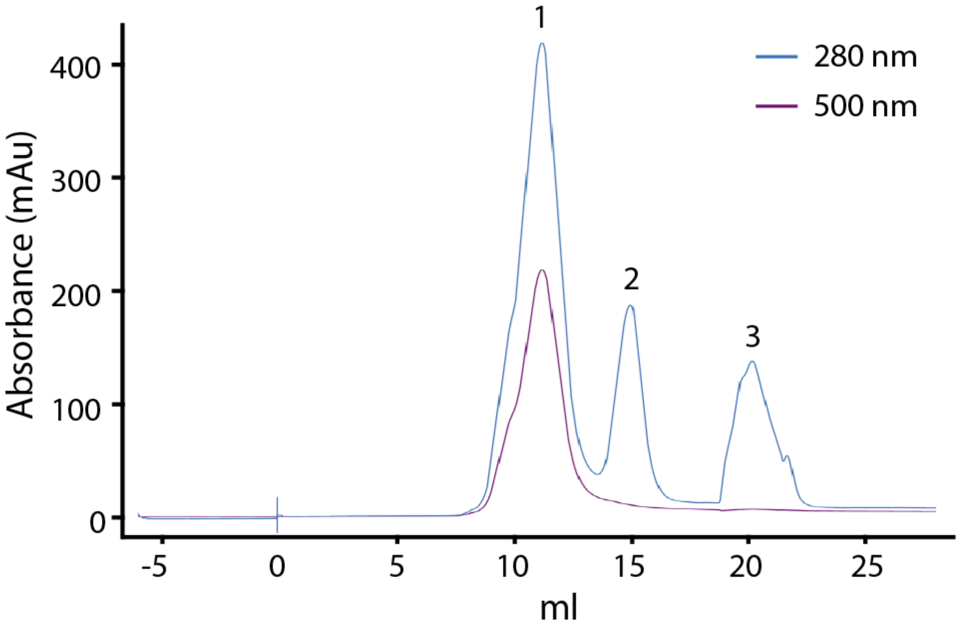

Figure S1. Size-Exclusion Chromatography (SEC) of the nanobody-gold conjugation. Superdex-75 increase column was used to separate 3 nm gold NPs conjugated to anti-mouse nanobody. Peak 1 is the conjugated nanobody and gold NPs, which show their absorbances at the wavelengths of 280 nm and 500 nm, respectively. Peak 2 corresponds to the unconjugated nanobody absorbing at 280 nm. And peak 3 corresponds to a small protein fraction after cleaving the His-Tag from the nanobody.

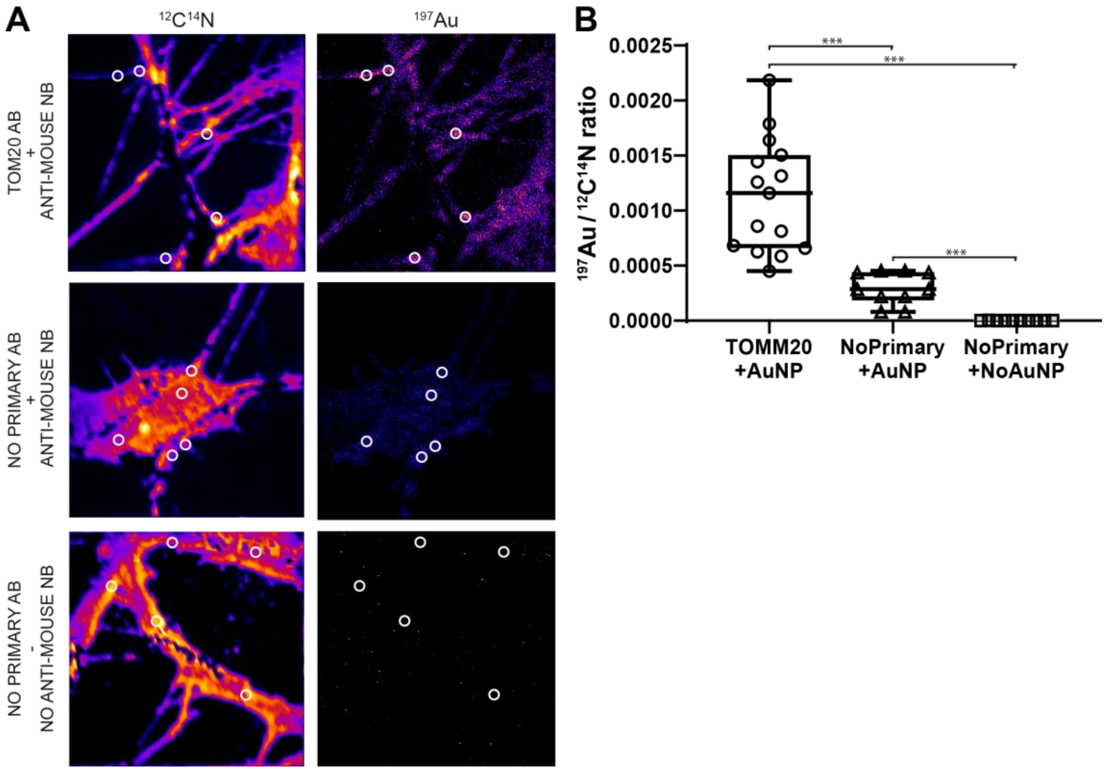

Figure S2. (A) Selection of ROIs. Representative example of how regions of interest (ROIs) were defined to compare the $^{197}Au/^{12}C^{14}N$ ratio between different conditions by selecting circular ROIs of 5 pixels diameter. **(B)** The chart shows a significantly higher signal of ^{197}Au signal in the neuronal cells labeled with anti-TOM20 primary antibody (AB) plus Au anti-mouse secondary nanobody (NB) compared to the negative control cells labeled with Au anti-mouse secondary nanobody in the absence of anti-TOM20 primary antibody, and negative control cells labeled with neither the anti-TOM20 primary antibody nor Au anti-mouse secondary nanobody. Each data point represents the average $^{197}Au/^{12}C^{14}N$ ratio value from all the pixels of individual ROIs (n=15 for labelled cells, n=10 for control cells). The statistical analysis was performed by the Kolmogorov-Smirnov tests (*** $p < 0.0001$). Error bars represent SEM.

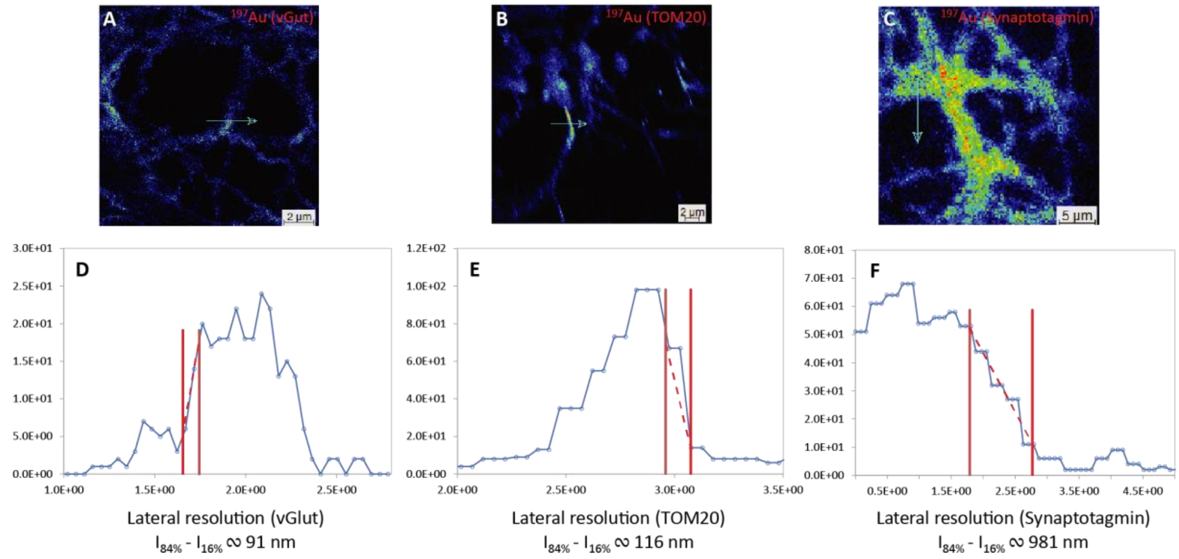

Figure S3. Line-scans on the ^{197}Au images of different structures in the hippocampal neurons labeled with the gold nanoprobes for determination of spatial resolution of NanoSIMS. **(A, B, C)** ^{197}Au images showing the structure of vGlut, TOM20, and Synaptotagmin. The line scan was performed along the arrow in each ion images. **(D, E, F)** Corresponding line scans for the structure of vGlut, TOM20, and Synaptotagmin.
